# Flexible Textile Strain Sensor Based on Copper-Coated Lyocell Type Cellulose Fabric

**DOI:** 10.3390/polym11050784

**Published:** 2019-05-02

**Authors:** Waleri Root, Tom Wright, Barnaby Caven, Thomas Bechtold, Tung Pham

**Affiliations:** Research Institute for Textile Chemistry/Physics, University of Innsbruck, Hoechsterstrasse 73, 6850 Dornbirn, Austria; tom.wright@uibk.ac.at (T.W.); barnaby.caven@uibk.ac.at (B.C.); thomas.bechtold@uibk.ac.at (T.B.); tung.pham@uibk.ac.at (T.P.)

**Keywords:** cellulose, electrical resistance, copper coating, electroless deposition, humidity sensor, strain sensor, lyocell fiber

## Abstract

Integration of sensors in textile garments requires the development of flexible conductive structures. In this work, cellulose-based woven lyocell fabrics were coated with copper during an electroless step, produced at 0.0284 M copper sulfate pentahydrate, 0.079 M potassium hydrogen L-tartrate, and 0.94 M formaldehyde concentrations. High concentrations led to high homogeneous copper reaction rates and the heterogeneous copper deposition process was diffusion controlled. Thus, the rate of copper deposition did not increase on the cellulose surface. Conductivity of copper coatings was investigated by the resistance with a four probe technique during fabric deformation. In cyclic tensile tests, the resistance of coated fabric (19 × 1.5 cm^2^) decreased from 13.2–3.7 Ω at 2.2% elongation. In flex tests, the resistance increased from 5.2–6.6 Ω after 5000 bending cycles. After repeated wetting and drying cycles, the resistance increased by 2.6 × 10^5^. The resistance raised from 11–23 Ω/square with increasing relative humidity from 20–80%, which is likely due to hygroscopic expansion of fibers. This work improves the understanding of conductive copper coating on textiles and shows their applicability in flexible strain sensors.

## 1. Introduction

Flexible conductive materials have been extensively investigated according to their promising potential applications in energy storage devices, photodetectors, pressure sensors, and light emitting displays [1,2,3]. The current research shows the feasibility of fiber/fabric coating to induce electrical conductivity. Coating of metallic copper layers on woven cellulose lyocell (CLY) fabrics through electroless deposition is important to achieve a large area sensor network for wearable smart textiles.

Cellulose is an important textile substrate due to its biodegradable, biocompatible, eco-friendly, non-toxic, and renewable properties [4]. It is chosen according to its high mechanical modulus property, a high strength and an inflexible crystallinity [5]. Several approaches are reported in the literature about how conductive substrates are implemented in large area fabrics. Flexible large area sensors are used for pressure and temperature sensing consisting of an organic coating on plastic and rubber substrates [6,7]. Huang et al. used a 150 cm^2^ cloth on a woolen band consisting of steel yarns for energy storage [8]. Shur performed the deposition of CdS/CuS films on 8 × 10 inches foils, which were suitable for application in e-textiles [9]. Yang et al. manufactured a carbon cloth of 160 cm^2^, which is embedded with Nickel nanoparticles through a metal-mediated pitting method [10]. Matsuhisa et al. printed elastic textile conductors of 30 × 20 cm^2^ size with silver nanoparticles which showed high conductivity after treatment [11].

Understanding the relationship between electrical conductivity and mechanical deformations of metal coated/electrical textiles remains under reported. Permanent stress and deformation of conductive textiles on textiles could lead to damages. It is essential to investigate the electrical resistance of conductive textiles during tensile strength and bending. Abry et al. investigated the damage in Carbon Fiber Reinforce Polymer (CFRP) by means of the electrical resistance [12]. Zhang et al. showed that knitted carbon fiber fabrics displayed greater changes in resistance under loading compared to stainless steel fabrics [13]. Lin et al. measured the electrical resistance during tensile loading on blended yarns consisting of polypropylene (PP) and polyethylene terephthalate (PET) coated with multi-walled carbon nanotubes (MWCNT) [14]. Perumalraj investigated the effect of the resistance on polyaniline (PANI) impregnated polyester/viscose, Bamboo, Bamboo/cotton and cotton fabrics [15]. Hansen et al. showed that Poly(3,4-ethylenedioxythiophene) and aliphatic polyurethane substrate retained conductive property during stretching and relaxation [16]. Kageyama et al. investigated a BaTiO_3_ impregnated carbon fabric, which indicated an electrical resistance during tensile tests [17]. The resistance of carbon nanotubes coated textile capacitor was studied by Yun et al. which investigated the behavior of resistance during mechanical strain in textile-based capacitors consisting of Carbon nanotubes coated fabrics [18]. Qu et al. reported the manufacture of extruded carbon fiber/polymethylmethacrylate composites, which increased their conductive property as the carbon fiber volume fraction for filaments of 1mm and 3mm diameter increased [19]. Won et al. explored copper nanowires with a high stretchable property for the application as stretchable electrodes [20]. Ali et al. detected the conductive property of cotton textiles impregnated with copper particles during an applied load as a function of elongation [21]. Nishio et al. used the four probe technique to measure the change of the electrical resistance of a woven fabric during cyclic elongation [22]. De Baere et al. measured the voltage in a carbon fabric composite by evaluating its electrical resistance [23]. Cui et al. investigated the electrical conductivity of cupro fabrics impregnated with silver nanowires during bending, shrinking and stretching [24]. Application in medicine, sports, energy storage [25] security and personal protection, military and fire departments are all suggested [26,27]. Conductive coatings on cotton textiles were used for sweat detection in a garment by measuring resistance [28] and for monitoring electrical signals of a plant during stimulation [29]. Zieba and Frydrysiak reported the response of resistance to elongation, strain or bending in a textile sensor for monitoring breathing [30]. Ehrmann et al. tested textile sensors in knitted fabrics for human breathing application by detecting the electrical resistance during elongation [31]. Li et al. investigated the increase of the length resistance of knitted conductive fabrics during extensile force [32]. In the review of Kongahage and Foroughi, conjugated double bond conductive polymers (CP) were described as promising materials for actuators consisting of polypyrrole (PPy), polyaniline (PANI) and PEDOT:PSS substrates. Those CP actuators can be used to monitor bending and linear motion [33]. Atalay et al. constructed a knitted textile strain sensor made of conductive yarns for human body motion detection [34]. In a different work, Atalay et al. used silver (Ag) plated nylon yarns for measuring the tensile strength [35]. Body motion was monitored by a strain sensors during resistance change based on a stretchable and conductive plain woven polyester fabric [36]. 

In summary, the above-mentioned works showed a successful implementation of conductive materials as metals (e.g., Cu, Ag), carbon substrates, conductive polymers (e.g., PET, PANI) to introduce an electric property in non-conductive substrates (e.g., PP, polyester, Bamboo, cotton). Using silver as conducive material in non-conductive substrates can be explained due to its high electrical conductivity of 6.3 × 10^7^ Sm^−1^ compared to 5.9 × 10^7^ Sm^−1^ of copper and 2 × 10^5^ Sm^−1^ carbon (graphite) substrates. Despite the lower cost of copper ($ 6.7/kg) compared to silver ($ 510 /kg), immediate oxide layer formation on the copper surface makes its application difficult [25]. The implementation of conductive materials in knitted fabrics and woven fabrics can be used in electronic components in textiles. An advantage of knitted fabrics is their good stretch ability, softness and durability, which makes their applications in strain/pressure sensors important. Woven fabrics were reported to be effective for the monitoring of the bending motion [26]. In the work of Ding et al., polyurethane (PU) nonwoven substrates were manufactured by electrospinning and were subsequently dip coated with conductive poly(3,4-ethylenedioxythiophene):poly(styrenesulfonate) (PEDOT:PSS) polymers. The PEDOT:PSS/PU substrates indicated a sheet resistance of 35–240 Ω/sq, withstand a strain of 200% and showed a constant resistance after 10 stretch release cycles [37]. Mahmood et al., showed the manufacture of conductive graphene oxide glass fiber substrates, which impart an electrical resistivity of 10^1^Ωm after electrophoretical deposition [38]. In the review, Ding et al. reported on the conductive sponges as pressure sensors like neat conductive sponges (e.g., impregnated with Cu nanowires, CNT), composite conductive sponges (e.g., composed of polyimide (PU), polyacrylonitrile (PAN)), conductive sponges impregnated with elastomers (e.g., silicone, polydimethylsiloxane (PDMS)) and like conductive material coated sponges (e.g., coated with carbon, Ag). The conductive sponges can be used for the human motion detection [39]. Baima et al. investigated fluoropolymer wrapped conductive threads in an electrical touch sensor assembly by using the triboelectric method known as the change of the surface potential resulted from the skin contact [40]. Ebrahimi and Gashti used PPy materials as electrical substrates in clay Ag-polypyrrole nanocomposites, which showed an enhanced electromagnetic shielding and conductive properties compared to untreated PPy materials after the photo reduction technique [41]. The use as wearable electronics to monitor different parameters from the environment such as temperature, pressure, moisture is clear.

Monitoring conductivity of electrical textiles through the resistance in a wet environment mimics the exposure of fabrics to rain and different humidity’s. Wet and dry treatments of textiles might lead to stiffness or reactivity changes [42,43]. Kramar et al. measured the electrical resistance of cotton and viscose fabrics impregnated with Ag^+^, Zn^2+^, and Cu^2+^ ions at different relative humidity conditions [44], apart from the change in resistance due to the ion treatment the resistance also decreased with increasing humidity. Shim et al. used carbon nanotubes coated cotton threads for humidity sensing and for mechanical/chemical durability tests [45]. The advantage of using copper as conductive substrate is its high conductivity/cost ratio in comparison to silver or nickel [25]. In previous work, copper layers were formed by electroless deposition on small woven CLY fabrics inducing conductivity [46]. To the authors best knowledge, there are few treatise reporting on electroless copper deposition (ECD) on a large (or easily scalable) area, i.e., 21 × 29 cm^2^ woven CLY fabric. Specifically, none dealing with the investigation of the influence of cyclic tensile tests, flex tests, extended water treatment for 18 h and different relative humidity on resistance. The aim of this work was to coat a 21 × 29 cm^2^ CLY textile surface with a copper layer during electroless deposition from aqueous solution and investigate its resistance as a function of fabric deformation. It included an optimization of chemical concentrations of CuSO_4_, formaldehyde and potassium hydrogen tartrate to achieve a strong adhesive and continuous copper layer. 

## 2. Experimental

Samples cut from 21 × 29 cm^2^ copper-coated CLY fabrics were used for investigation of resistance change during extended water treatment times, different relative humidity, flex and cyclic tensile tests. The four probe technique was used to monitor the resistance during different relative humidity (RH), during tensile testing and after flex tests. For humidity tests, a climate chamber was used at 25 °C for 6 h.

### 2.1. Materials

The chemicals were used as received. Potassium hydrogen L-tartrate (C_4_H_5_KO_6_ 99 wt %) was purchased form Fulka (Buchs, Switzerland). Tin(II) chloride × 2 hydrate (SnCl_2_ × 2H_2_O) and formaldehyde (H_2_CO 36.5 wt %) were received from Riedel-de-Haen (Seelze, Germany). copper sulphate pentahydrate (CuSO_4_ × 5 H_2_O 99.5 wt %), ethanol (C_2_H_6_O > 99.8% with ca. 1% Methyl Ethyl Ketone) and ammonia (NH_3_ 25 wt %) were purchased from Carl ROTH GmbH + Co. KG (Karlsruhe, Germany). Sodium carbonate (Na_2_CO_3_ 99 wt %) was obtained form MERCK (Darmstadt, Germany). Silver nitrate (AgNO_3_ 99.9 wt %) was obtained from VWR PROLABO (Leuven, Belgium). Sodium hydroxide (NaOH 50 wt % solution) was obtained form Deuring GmbH & Co KG (Hörbranz, Austria). The woven cellulose lyocell fabrics were kindly provided by Lenzing AG (Lenzing, Austria), which is a plain woven cellulose lyocell fabric (143 g/m^2^ weight, fibers of 1.3 dtex linear density and 39 mm length, 40 warp and 31 fill threads per cm). Filter papers (MN 615 ¼, 70 g/m^2^, thickness 0.16 mm, surface smooth, ash content 0.1%, α-Cellulose content 95%, average retention capacity 4–12 µm) of 110 mm diameter were obtained form MACHEREY-NAGEL GmbH/Düren/Germany.

### 2.2. Electroless Copper Deposition on A4 Sized Fabrics

The CLY fabrics (A = 21 cm × 29.7 cm = 623.7 cm^2^ m = 5 g) were cleaned with 1.35 M NaOH, 20 mL H_2_O de-ionized (DI) solution for 10 min. The surface of the CLY fabric was rinsed with 50 ml H_2_O (DI) and dried at ambient conditions for 24 h. The CLY fabric was dipped into 19.8 M EtOH (MEK) 100 mL and 0.029 M SnCl_2_ × 2H_2_O solution for 1 min. Afterwards, it was dried at ambient conditions for 1h. The CLY fabric was immersed into a 0.042 M AgNO_3_, 100 mL H_2_O (DI) and 2.64 mL (1.43 M) NH_3_ solution for 1 min at pH 11.58. The CLY fabric was dried at ambient conditions for 24 h. Different concentration of C_4_H_5_KO_6_, CuSO_4_ × 5 H_2_O, NaOH, H_2_O (DI) and CH_2_O were used. Constant contents of 0.019 M Na_2_CO_3_ and H_2_O (DI) were used in all experiments. As shown in Table 1, all experiments were performed for 3h at pH 12.5 and room temperature.

### 2.3. Flex Test

The characteristics of fatigue due to cyclic bending of copper-coated CLY fabric strip were determined with an adapted method according to DIN 53 543:1979-02, DIN EN ISO 17707:2005-10 and EN ISO 20344:2004-10 standards. A Cu-coated strip with a size of 1.5 cm × 20 cm was attached to the permanent bending machine and was exposed to a total number of 6000 bending cycle. All bending tests were performed at a bending rate of 180 cycle/min. The top and bottom part of the copper-coated CLY fabric strip was fixed with two jaws. 10 cm length of the copper-coated fabric strip were suspended to permanent bending at bending apparat (Biegeprüfmaschine (BPM), Pirmasens, Germany). The angle of friction change from 111° to 179° during flex tests.

### 2.4. Resistance During Cyclic Elongation

The sheet resistance of copper-coated CLY fabrics was investigated during repetitive elongation in relation to the ASTM D 257-07 standard. The copper-coated CLY fabric strips had the size of 1.5 × 19 cm^2^ and were placed between jaws with a rubber isolation layer at a grip to grip separation of 100 mm. Metallic copper strips monitored the resistance of the copper-coated CLY fabrics. The fabric was elongated and relaxed during permanent elongation cycles (Zwick/Roell/Ulm/Germany (Z010 test Control II)). The preload was set to 5 N with 50 mm/min speed and 60 sec time. The standard force increased from 2.24 N, 10 N, 20 N, until a maximum of 30 N and decreased back to 20 N, 10 N, 2.24 N at a speed of 100 mm/min. Measurements were taken at a constant current of 20 mA applied by the power supply and voltage was monitored with a multimeter during fabric deformation tests. The two inner strips recorded the voltage during each load for 30 s and the resistance (*R*) was evaluated according to the equation below:

*R* = (*U* × *w*)/(*I* × *l*) (1)

where *U* is the measured voltage, *I* the applied current, *w* the width of the Cu fabric strip and *l* the investigated distance between the electrodes. During extended water treatments, the resistance of copper layer on CLY fabrics was measured with a multimeter (FLUKE) in a 100 mL H_2_O (DI) solution stirring at 750 rpm for 18 h. 

### 2.5. Electrical Resistivity Tests in Fabrics

The electrical resistivity of dry fabric was measured with METRISO/3000 resister tester (Wolfgang Warmbler/Eppingen/Germany) for silver seeded CLY fabrics relating to the DIN 54345 standard. The values were recorded after one minute at 505 V and the measurements were repeated for three times. 

### 2.6. Four Probe Technique Measurements

The sheet resistance (*R_sh_*) was measured in a coaxial arrangement with a four point probe technique at a spacing of 1cm between the electrodes. The resistance was evaluated according to the equation below:

*R*_*sh*_ = *4.532* × (*U*/*I*) (2)

where *U* is the measured voltage and *I* the applied current. All measurements were related to the ASTM standard F1529-94.

### 2.7. Copper and Silver Determined with AAS (Atomic Absorption Spectroscopy)

50 mg of the copper-coated fabric was cut from a 5 g CLY fabric and was immersed into a (15 wt %) nitric acid (HNO_3_) solution (30 mL) at 80 °C for 2 h. The extraction solution was filtered with a filter paper (d = 110 mm) for 5 min. A 10 mg/L stock solution of Ag^+^ (0.46 × 10^−3^ M AgNO_3_) and 10 mg/L of Cu^2+^ (0.16 × 10^−3^ M CuSO_4_ × 5H_2_O) were used to prepare calibration solutions in the range of 0–2 mg/L for Cu^2+^, 0–1.5 mg/L for Ag^+^. The flasks (100 mL) and tubes (50 mL) were rinsed with (6 wt %) HNO_3_ before AAS measurement. Silver (Ag) and copper (Cu) contents in CLY fabrics were determined with AAS (contrAA 300, Analytic, Jena Germany) in an air-acetylene flame with a 100 mm burner at a wavelength of 328.1 nm for Ag and of 324.8 nm for Cu. Linear regression of the calibration standards and the sample absorbance were used for the metal content detection in CLY fabrics. The metallic content was obtained due to Equation (3) in CLY fabrics. The analysis was related to DIN 38404 Teil 18, EN ISO 17294-2 and DIN 38406 Teil 7.

*Cu_f_* = (*Cu_s_* × *V*)/*W* (3)

where *Cu_f_* is a copper content in fabric (mg/g), *Cu_s_* is a Cu content in dilute solution (mg/L), *W* is the mass of the investigated fabric piece (g) and *V* is a volume of extraction solution (mL).

### 2.8. Topology Investigations

The environmental scanning electron microscopy (ESEM) and energy-dispersive X-ray spectroscopy (EDX) were conducted with the JEOL JSM-7100F microscope, which was operated in the range of 10–20 keV. The copper-coated CLY samples were placed on an aluminium specimen with a conductive carbon layer on top without any coating. All machine operating are given specific parameters in the figures. The topology was investigated with a laser scanning microscope (VK-X100 series LSM 3D Profile Measurement from KEYENCE). The images were recorded with a 100× lens at a working distance of 4.7 mm. The samples were not under tension and were placed on a paper sheet during measurements. The topology of copper-coated CLY samples was characterized with Laser Scanning Microscopy (VK-X100 series LSM 3D Profile Measurement, KEYENCE, Tokyo, Japan). All FTIR measurements were conducted with the ATR-FTIR device (Vector 22, Bruker, Karlsruhe, Germany).

## 3. Results and Discussion

### 3.1. Electroless Copper Deposition on 21 × 29 cm^2^ Cellulose Fabrics

Table 2 shows a summary of results of CLY fabrics produces at different molar concentrations of CuSO_4_, H_2_CO and C_4_H_5_KO_6_ during the ECD. The silver content [Ag] on the cellulose substrate indicates a high resistance (*R*) due to silver seeds, which are not connected to one another. The copper-coated fabrics (CU 1, CU 2, CU 3) indicated different copper contents [Cu], roughness along the line values (*S_h_*), sheet resistances (*R_sh_*) and sheet conductance values (*G_sh_*). The Cu1 sample showed the lowest sheet resistance, lowest copper content and the highest sheet conductance comparing to Cu2 and Cu3 samples. 

In Table 2, sample CU 3 showed a coated and uncoated copper surface, although the amount of copper was high due to the high Cu concentrations in solution. The overall deposition rate (dCu^0^/dt) of the electroless copper deposition can be explained by Equation (4). The first term describes a heterogeneous copper deposition rate (*dCu*^0^/*dt*)*_het_* on the textile surface (*A*) and the second term shows a homogenous copper reaction rate (*dCu*^0^/*dt*)*_hom_* in solution.

(4) $$\frac{dCu^{0}}{dt}={\left(\frac{dCu^{0}}{dt}\right)}_{het}+{\left(\frac{dCu^{0}}{dt}\right)}_{hom}$$

The heterogeneous copper deposition rate depends on the $\left[OH^{-}\right]$ hydroxyl, $\left[Cu^{2+}\right]$ ion, [Tartrate] complexing agent, $\left[CH_{2}O\right]$ formaldehyde concentrations and the textile surface (*A*) as is shown in Equation (5).

(5) $${\left(\frac{dCu^{0}}{dt}\right)}_{het}=k_{het}\times \left[OH^{-}\right]\times \left[Cu^{2+}\right]\times \left[CH_{2}O\right]\times A\times 1/\left[Tartrate\right]$$

The homogeneous copper reaction rate in solution does not depend on the textile surface (*A*), which is shown in Equation (6).

(6) $${\left(\frac{dCu^{0}}{dt}\right)}_{hom}=k_{hom}\times \left[OH^{-}\right]\times \left[Cu^{2+}\right]\times \left[CH_{2}O\right]\times 1/\left[Tartrate\right]$$

where $k_{het}$ and $k_{hom}$ are kinetic constants for heterogeneous and homogenous reactions.

**Figure 1:**
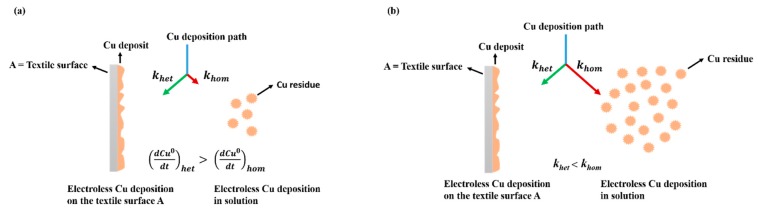
A schematic drawing of the electroless copper deposition during low (**a**) and high (**b**) Cu^2+^ concentrations in solution.

At low OH^−^, Tartrate, Cu^2+^ and CH_2_O concentrations in solution, the overall copper deposition reaction rate is slow and occurs mainly on the textile surface as highlighted by the green arrow (Figure 1a). The heterogeneous copper deposition rate is large comparing to the homogenous copper reaction rate, which leads to a slow reaction in solution (red arrow). 

At high OH^−^, Cu^2+^, Tartrate and CH_2_O concentrations in solution, the heterogeneous copper deposition rate on CLY surface (*A*) competes with the homogeneous copper reaction rate in solution (Figure 1b). The diffusion of Cu^2+^ ions to the textile surface (a) limits the copper deposition rate. Thus, the formation of copper residue occurs in solution simultaneously to the copper deposition on the textile surface (*A*) and the overall copper deposition rate is large. 

**Figure 2:**
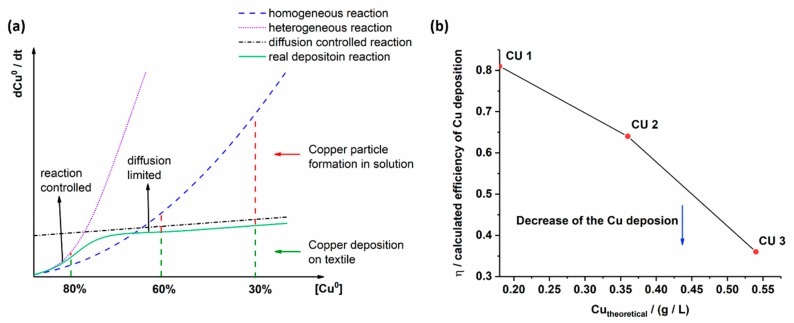
Theoretical (**a**) and experimental work (**b**) of the copper deposition on the textile surface.

An adaptation of the overall Cu^0^ deposition rate will be necessary if the Cu^2+^ concentration changes. Figure 2a,b depicts a theoretical and experimental work of the electroless Cu deposition. 

At low chemical concentrations (Figure 2a, the reduction and deposition of metallic copper on the silver seeded textile surface depends on Cu^2+^ ion diffusion in solution (diffusion controlled reaction). The reduction of Cu^2+^ to Cu^0^ near the surface (*A*) is fast (heterogeneous reaction) and for the copper layer formation Cu^2+^ ions diffuse from solution to the silver seed textile surface where reduction to Cu^0^ occurs. Thus, a copper layer grows continuously by contributing more Cu^2+^ ions from solution (real reaction), which explains the large amount of copper (80%) detected on the silver seed textile surface. Side reaction as copper deposition on the beaker walls and the not reduced Cu^2+^ ions in solution are not pronounced. Increasing chemical concentrations (Cu^2+^, C_4_H_5_KO_6_, CH_2_O, OH^−^) lead to pronounced reduction rate and copper particle formation in solution (homogeneous reaction). As a result, the amount of copper on the textile surface decreases (60%). The formation of copper particles refers to the nucleation in solution, which compete with silver seeds on the textile for the Cu^2+^ ion consumption. At high chemical concentrations (Cu^2+^), nucleation and growth of copper particles in solution dominate over the copper deposition on the textile surface. The heterogeneous deposition of copper at the textile surface enters into a diffusion controlled reaction, thus further increase in Cu^0^ formation does not occur (30%). The Cu^0^ formation in the bulk solution however gain importance. The plots were made with regard to various simplifying assumptions such as a constant volume of H_2_O (DI), beaker shape and textile surface during the experiments. In Figure 2b the calculated efficiency *η* of the copper deposition is high at low Cu^2+^ concentrations (CU 1). It drops for CU 2 and CU 3 samples at high Cu^2+^ concentrations. Based on the conditions in CU 1 an enhancement of copper deposition on textiles can be worked out by adding small OH^−^, Cu^2+^ and CH_2_O concentrations to the solution over time. The theoretical work corresponds well to the experimental work of CU 1, CU 2 and CU 3 samples manufacture.

**Figure 3:**
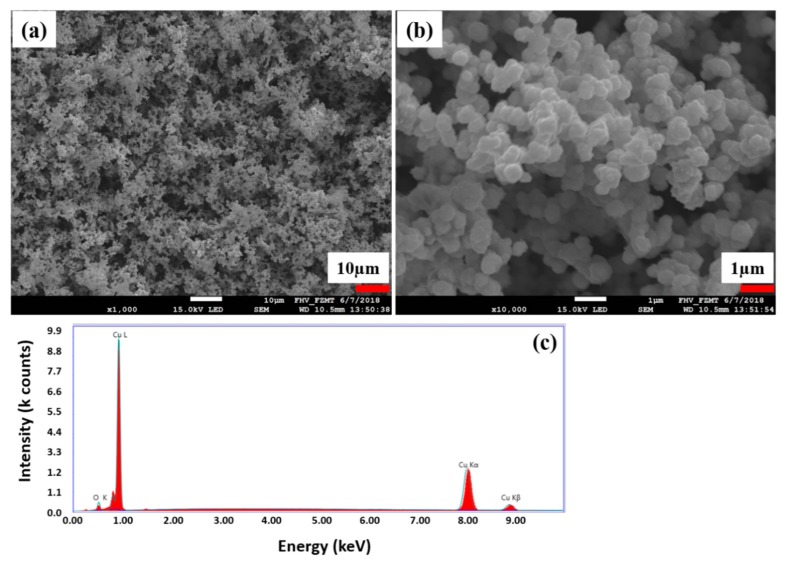
Fragile copper residue (**a**,**b**) formed during the electroless copper coating of fabric (CU 2) is investigated with EDX (**c**).

Apart from the copper coating deposition at the silver seeded CLY fabric, high Cu^2+^ concentrations (0.0568 M, 0.0852 M) enhanced the kinetic reaction for the copper residue formation in solution (Figure 3). At high Cu^2+^, formaldehyde and C_4_H_5_KO_6_ concentrations, copper residue of 1 µm formed in solution, which is shown in Figure 3b for the CU 2 sample. According to EDX (Figure 3c), the copper residue shows peaks of higher relative intensity for copper with respect to the oxygen peak, which may indicate the formation of copper particles in solution.

Due to the lowest sheet resistance and continuous copper layer, the Cu1 sample was chosen for further investigation in mechanical tests.

**Figure 4:**
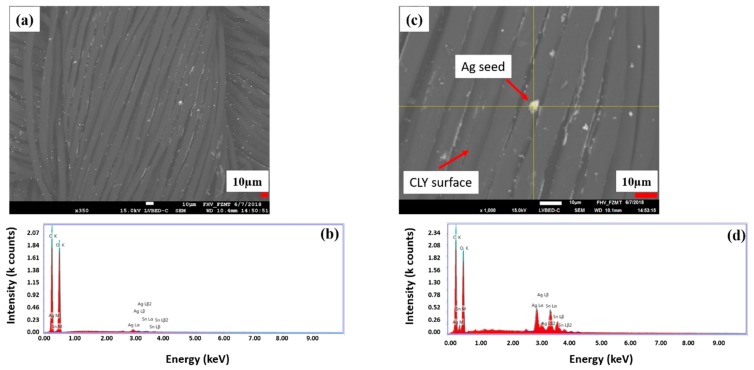
The surface of a silver seeded CLY (CU 1) sample is investigated with SEM (**a**) and EDX investigation (**b**). The magnification a silver seed (**c**) is shown, which is investigated with EDX (**d**).

Figure 4a,b shows a random distribution of the silver seeds on CLY fabric, which build a catalytic surface for the electroless copper deposition. Relative intensity peaks of Ag and Sn are high when the EDX investigation is focused on a single silver seed (Figure 4c,d) comparing to the yarn surface. High relative intensity peaks of C (carbon), O (oxygen) species resulting from the cellulose substrate. Sn^2+^ reduced Ag^+^ to Ag^0^ (metallic) from aqueous solution and formed a silver seed as catalytic surface on the CLY fabric. No continuous Ag layer was observed on the CLY surface.

The silver content in Table 2 is too high to be related only to silver seed of 8 µm size (Figure 3c) and Figure 4c shows silver seeds smaller than 8 µm. The silver seeds had different sizes and were randomly distributed on the CLY surface. In Table 2, the content of silver [Ag] is lower in comparison to the copper content. Thus, silver seeded CLY fabrics indicated a high resistance in GΩ (Giga Ohm) range, which drops to 1.89 Ωsq^−1^ and 5.24 Ωsq^−1^ after the ECD. The copper-coated CLY fabrics reveal a conductive property due to the copper coating. 

### 3.2. Topological Investigation of the Copper Coating

**Figure 5:**
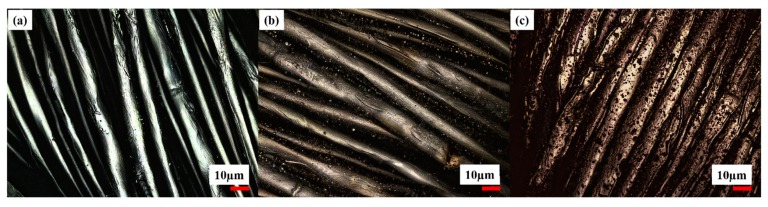
Topology investigation of uncoated (**a**), silver seeded (treated at 0.042M AgNO_3_) (**b**), and copper layer coated (**c**) CLY CU 1 sample.

Figure 5 indicates topological investigations of untreated (a), a silver seeded (b) and copper-coated CU 1 (c) samples.

**Figure 6:**
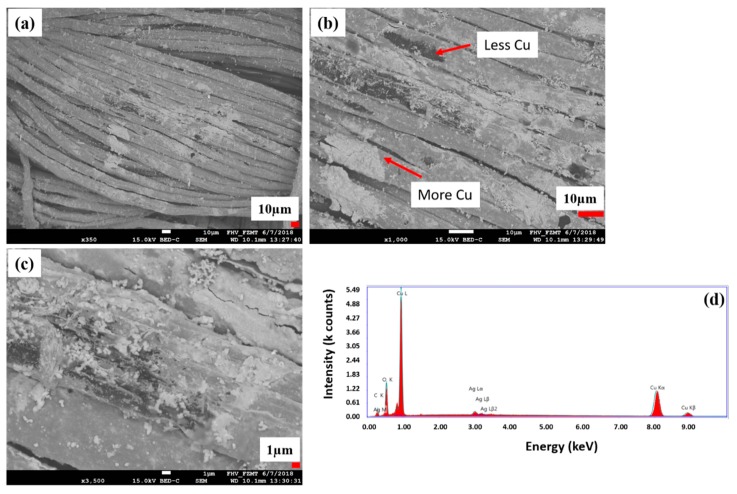
Topology of the copper-coated CLY (CU 1) fabric investigated with SEM (**a**–**c**) and EDX (**d**). High copper amount appears in bright color and low copper amount in dark color.

The topology investigations showed a gray image of untreated (Figure 5a), a bright yellow image after silver seeding (Figure 5b) and dark red copper-coated image of the CLY fabric (Figure 5c). For additional topological investigation, the copper-coated CLY CU 1 sample is investigated with SEM/EDX (Figure 6). Bright areas consists of a higher copper density in comparison to dark areas. Copper islands (bright areas) grow on a continuous copper layer (dark areas) as highlighted in Figure 6a,b. The surface might contain impurities like copper oxides, or organics due to EDX spectra (Figure 6d). Besides Cu, O and C elements were detected, which were related to the CLY.

Figure 6c depicts copper islands smaller than 1 µm, which could be explained by a staggered layer growth during the ECD. Copper might grow in two directions as a copper island (bright color) by increasing its thickness and as a layer (dark color) covering the cellulose surface. 

Table 3 is related to the EDX spectra in Figure 6a,d, which shows the elemental distribution of Cu, O, C and Ag from the copper-coated cellulose surface (CU 1). It indicates that the Cu element has the highest weight proportion of 81.36% compared to the O element of 9.46%, C element of 7.4% and Ag element of 1.77% on the copper-coated cellulose sample.

### 3.3. Cross-Section and Copper-Coated Lyocell Yarn

**Figure 7:**
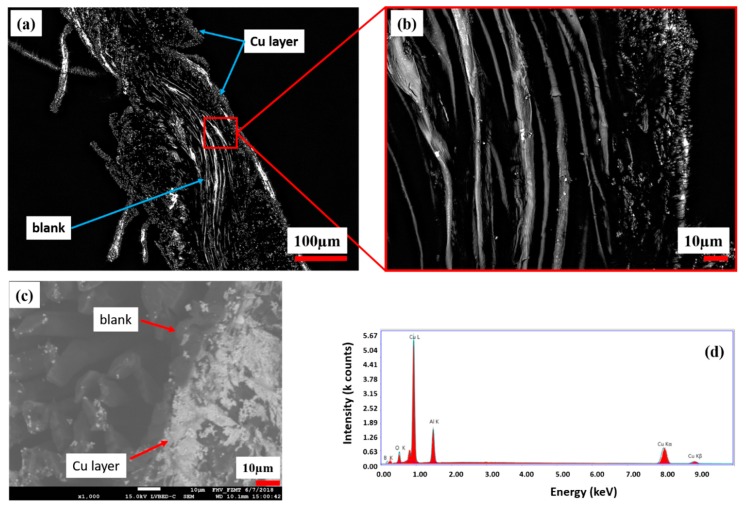
Cross section of the copper-coated CLY fabric (CU 1) and its magnification (**a**,**b**) captured with LSM. SEM/EDX investigation of the same cross section are shown in (**c**,**d**).

In Figure 7, a cross-section of the copper-coated CU 1 sample is investigated. According to the topological LSM and SEM investigations, the CU 1 sample shows less copper amount (bright color) in the interior side comparing to its outer surface (Figure 7a,c).

In Figure 7c, bright areas are related to the copper layer, which is formed on the outer surface of the CU 1 cross section. Figure 7d indicates high relative intensity Cu peaks comparing to O peaks, which are recorded from the cross section. The appearance of Al and B peaks resulted from the Al table as unwanted side effects and could not be excluded during measurement. There was no continuous copper layer observed in the interior side of the CLY cross section. The Cu deposition occurred on the silver seeded CLY fabric surface and stagnated in the interior side due the low accessibility of solution.

**Figure 8:**
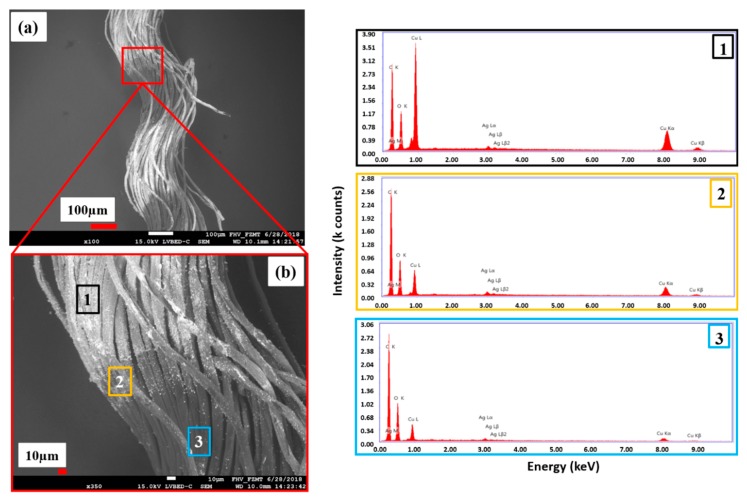
SEM/EDX investigations of the copper-coated CLY (CU1) yarn (**a**) show three different coating areas (**b**) (1, 2, 3).

One single yarn was separated from the copper-coated CU 1 CLY fabric and its topology was characterized with SEM/EDX (Figure 8a). Figure 8b shows three copper areas on the CLY yarn appearing in bright color for the copper coating (area 1), in grey for an intermediate coating (area 2) and in dark for non-coated area 3. Each area was investigated separately with EDX, which revealed different copper amounts. Different copper coatings are related to the yarn directions in warp and weft on the CLY sample. 

During the ECD, the copper species was differently distributed on the CLY fabric in an unstirred solution. Consequently, three different areas of copper species (areas 1, 2, 3) appeared on the CLY yarn (Figure 8b). This can be explained by different copper diffusion rates from the bulk to the surface and in the inner part of the CLY fabric. The Cu^2+^ ions diffused from solution and were reduced to Cu^0^ on the silver seed surface of the CLY leading to a copper coating. The diffusion of Cu^2+^ ions to the interior part of the CLY fabric was less efficient, which lead to no copper coating. According to the EDX spectra, copper peaks of low relative intensity were detected in area three (Figure 8) in comparison to area one and two. The relative intensity of copper peaks decreased from area one to area two and reached its lowest value in area three. Ag peaks from the Ag seeds were recorded at a low relative intensity comparing to the copper peaks while the C and O relative intensity peaks were similar in all EDX spectra.

### 3.4. Mechanical Testing of the Copper-Coated Lyocell

**Figure 9:**
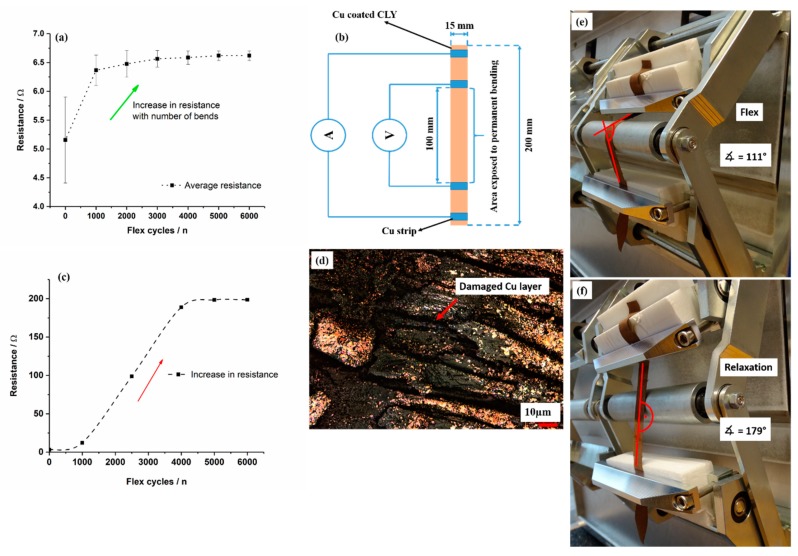
The change of resistance after flex tests (**a**) is measured with the four probe method (**b**). and with the two probe method (**c**). A damaged Cu layer of the CLY strip (CU 1) (**d**) is recorded with LSM after flex tests at different bending angles (**e**,**f**).

To design spatial copper conductor lines across the garment, one must take into account the critical places like knees and elbows. Such places are highly vulnerable with regard to bending and load during movement. Continuous flex cycles might cause permanent deformation of the garment, which could lead to a damaged copper layer and a loss of an electrical signal. The copper-coated CLY strip was subjected to continuous flex cycles around a cylinder of 15 mm radius in a flex test. During the flex cycles, the fabric did not come under tension. Therefore it is essential to investigate the sheet resistance of the copper layer after flex tests. Figure 9a depicts the results after different flex cycles of the copper-coated strip. 

The resistance of the copper-coated CLY strip went up from 5.1 Ω before flex cycles to 6.5 Ω after 2000 flex cycles. It approached a plateau of 6.6 Ω/1500 mm^2^ from 4000 to 6000 flex cycles. The large error bars at zero flex cycles can be explained with the different local resistances of each copper-coated strip. After 1000 flex cycles, the topology indicated a damage of the copper coating, which increased the resistance (Figure 9d). The copper deposit broke apart after 1000 flex cycles by creating cracks in the coating. Due to the low change of resistance with extended flex cycles no further damage of the copper layer was observed. During flex cycles the angle of bending changed from 111° to 179° (Figure 9e,f) when the copper-coated strip was bended by a metallic cylinder. The resistance was measured after each 1000 flex cycle when the copper-coated CLY strip was placed on an isolating paper background without applying pressure during measurement (Figure 9b). In Figure 9c, the resistance was measured with a two probe method during flex tests and approached a constant value of 200 Ω after 6000 bending cycles. Abry et al. investigated a carbon fiber epoxy substrate, and obtained different electrical resistance [12]. In their post-buckling tests, the applied compressive load to carbon fiber differs to this work, in which a copper coating is investigated on non-conductive cellulose material.

### 3.5. Cyclic Elongation of the Copper-Coated Lyocell

**Figure 10:**
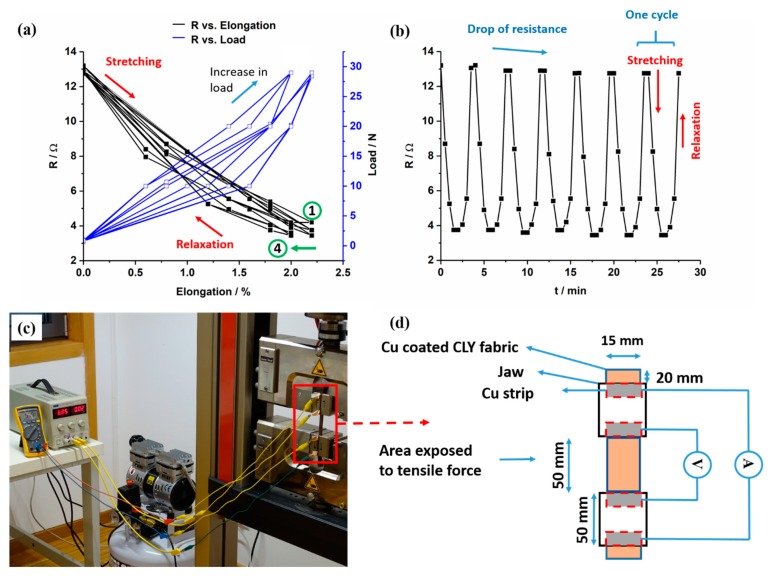
The change of resistance *R* (Ohm) of the Cu-coated CLY fabric (CU 1) (d = 15 mm, l = 50 mm) and applied load are recorded as a function of elongation (**a**) and time (**b**). The detection of the resistance change was conducted with the four point technique (**c**,**d**).

In Figure 10, the resistance (*R*) of the copper-coated CLY fabric changed as a function of cyclic elongation. The copper-coated CLY fabric was elongated and relaxed in a cycle, which was fixed between two jaws with an isolating rubber layer. The resistance change during elongation of Cu-coated CLY strip of 28.5 cm^2^ was investigated with the four point method. During cyclic elongation, current was applied by the two outer Cu strips while measuring the voltage with two inner Cu strips (Figure 10c,d). 

The decrease and increase in resistance of the copper-coated CLY strip are investigated during cyclic elongation marked by black curves in Figure 10a. Blue curves show the elongation and relaxation of the copper-coated strip caused by an applied load. The resistance of the copper-coated CLY strip decreased during elongation and reached a stable value of 2% after 4 cycles as marked by a green arrow. Numbers one and four label those cycles. The contact between Cu-coated yarns influenced the resistance of the CLY strip during elongation. During elongation, the Cu-coated CLY fibers are smashed by an applied load leading to an enlargement of the contact area and to a decrease in resistance. At relaxation, the resistance increased due to separation of the Cu-coated CLY yarns caused by a lower tensile force. Figure 10b depicts a steady decrease of the fabric’s resistance with applied tensile force and an increase with relaxation. It is assumed that the applied tensile force squeezed the copper-coated yarns closer together by decreasing their distance. Thus, the copper-coated yarns were oriented in the direction of the applied tensile force, which caused a decrease in resistance during elongation. During relaxation, the resistance of the copper-coated cellulose increased upto 13.2 Ω, which can be explained through the small fabric’s elongation of 2%. The purpose for conducting the elongation test up to 2% was to investigate the suitability of the copper-coated cellulose fabric to be used in a respiration sensor. Since the movement of the human body during breathing does not have high strains, the respiratory sensor can be implemented in a garment to monitor breathing. The elongation of 2% is investigated on the inelastic cellulose material and is related its small thickness. After four cycles, the resistance went up and reached a constant value of 13 Ω during further cycles. It is assumed that some copper fibers did not relax after four cycles. Long-time cyclic tests are predicated to show a constant resistance over time due to the smashed state of the copper-coated cellulose fibers during elongation. The resistance of the copper-coated cellulose fibers is expected to increase due to abrasion and damage of the copper coating during extended durability test, resulting in the sensitivity loss of the strain sensor. We expect the behavior of the Cu-coated cellulose similar to the PEDOT:PSS/PU sample as reported in the work of Ding et al. [37]. The results emphasize the potential of copper-coated CLY fabrics for strain sensors by monitoring textile deformation on a large scale (e.g., during breathing). Zhang et al. used stainless steel and carbon knitted fabrics during cyclic strain tests, whose work showed higher values of resistance during cyclic elongation [13]. In their experimental set-up, high strain and high resistances were related to a loosen sample structure comparing to a woven copper-coated cellulose in this work. Abu-Khalaf et al. produced stretchable circuits composed of PDMS (polydimethylsiloxane) and silver nanoparticle inks, which showed a resistance of 800 Ω during stretching of 25% at 45° [47]. In this work, a copper coating covered the surface of non-conductive cellulose.

### 3.6. Copper-Coated Cellulose as Moisture Sensor

**Figure 11:**
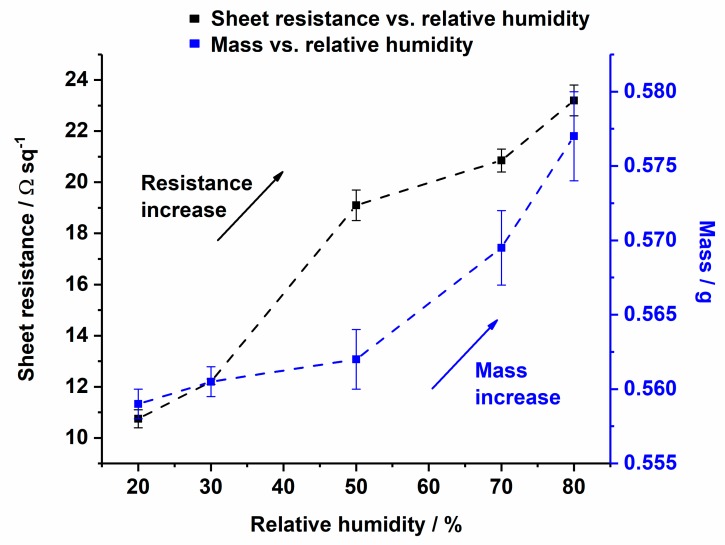
Change of sheet resistance (from 10.8 Ωsq^−1^ to 23 Ωsq^−1^) and mass change (from 0.55 g to 0.58 g) of the Cu-coated CLY (CU 1) fabric as a function of relative humidity.

In Figure 11, the resistance and the weight of a copper-coated CLY were investigated as a function of RH. The change of the RH was measured between 20% and 80% in a climate chamber in air at 25 °C. The copper-coated fabric was preconditioned for 2 h in a climate chamber at 20% RH and 25 °C. Subsequently, the change in RH was recorded stepwise at 30%, 50%, 70% and 80% RH. A period of acclimatization was needed between the steps for monitoring the fabric’s weight. Using the mass balance for weight monitoring, allowed the time to be determined when the fabric´s weight did not change leading to an equilibrium. After equilibrium, the resistance of the copper-coated fabric was measured with a four point technique and the RH was set to the next step. Its resistance and mass raised with the increase in RH, which could be explained due to hygroscopic swelling of the cellulose yarns. An increase in the fabric’s resistance might be explained due to the disruption of the current along the copper layer caused by hygroscopic expansion of the CLY fabric. During hygroscopic expansion of the CLY fabric, the copper coating was damaged between fibers and current can flow through a smaller copper coating area, which increased the resistance.

Kramar et al. investigated the decrease in electrical resistance of cotton and viscose impregnated metal ion fabrics from Giga Ohm to the Mega Ohm magnitude [44]. Their material was not conductive due to the impregnation of metal ions, which differs to the conductive copper coating in this work.

### 3.7. Durability in Water

**Figure 12:**
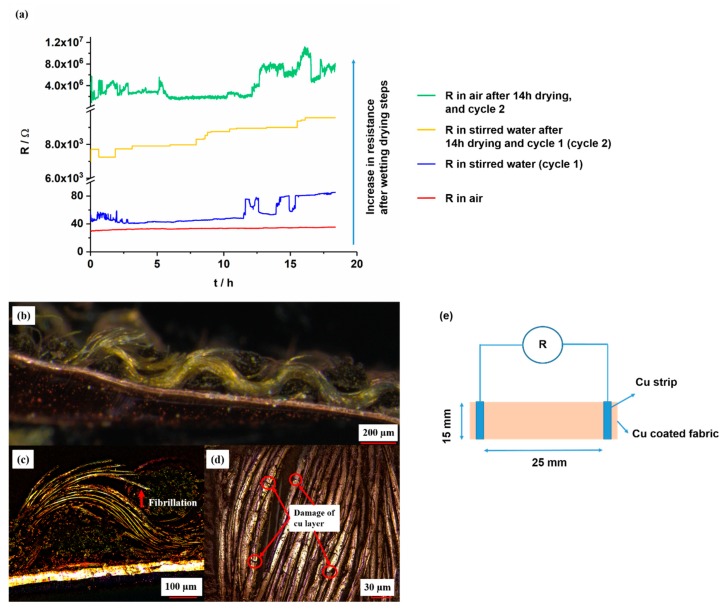
Resistance change of Cu-coated CLY (CU 1) fabrics (d = 15 mm, l = 25 mm) measured in air (red and green curves) and during stirring in water at 25 °C at a stirring speed of 750 rpm (blue and orange curves) (**a**). The figure shows a connection between Cu-coated CLY and Cu foil (**b**), the fibrillation of Cu-coated CLY fibers (**c**), the damaged copper coating (**d**) and the measuring procedure (**e**).

The resistance (*R*) change of the copper-coated CLY strip was recorded as a function of time (*t*) in air and in stirred H_2_O (DI) solution for 18 h. It was measured with a two probe technique by using a Handheld LCR Meter. In air, it indicates a value of 35 Ω in comparison to the resistance in water of 53 Ω/375 mm^2^ (Figure 12a). The aim of this work was to investigate the stability of copper-coated layer on CLY fabrics during repeated wetting/drying cycles. After first wetting (blue line) in H_2_O (DI), the electrical resistance was recorded over a time period of 18 h and raised from 53 Ω to 85 Ω comparing to the measurement in air. The copper-coated CLY fabric was dried for 14 h and immersed in to the second H_2_O (DI) solution for further 18 h. Its resistance went up from 6 kΩ to 9.5 kΩ, which might result from swelling and loosening of CLY fibers during wetting and drying. During the second measurement in air, the resistance of the copper-coated layer increased from 4 MΩ to 8 MΩ. The repeated wetting and drying steps destroyed the copper layer on the CLY fabric due to the high resistance. Movement of non-coated fibers due to the inside swelling might damage the Cu layer on the surface. Oxidative corrosion might destroy the copper layer, which was not observed after the experiment due to the Figure 12b. Thus, the copper layer could be damaged by the mechanical deformation of the fabric, which led an increase in resistance. The Cu-coated CLY has a wavy structure, which makes monitoring of the resistance (*R*) in water challenging due to the plain structure of the Cu strip electrodes (Figure 12b).

Fibrillation of copper-coated CLY fibers from the yarn might lead to a decrease of the conductive contact area (Figure 12c). The decrease of the contact area between copper-coated fibers raised the electrical resistance during swelling. Swelling of cellulose fabric separated the copper-coated fibers in aqueous environment during which the CLY fabric was soaked with water. During drying in air, the water content evaporated from the copper-coated fabric, and the copper-coated fibers remain in a loosen state. Long term water treatment caused damage in the copper coating and increased its resistance from 38 Ω to 16 MΩ, which was observed after second drying step (Figure 12d). The copper-coated fabrics remained conductive after first wetting in water (DI) which can be explained through a good adhesion between the copper layer and the CLY substrate. Thus the cellulose material could be incorporated in smart textiles for an extended operation time during wet weather (e.g., raining). No peeling of the copper layer was observed during resistance measurement in water. As an alternative, a PES substrate can be used instead of cellulose to avoid swelling. The copper-coated CLY fabric was not a plane substrate, which was connected to the Cu strip through its wavy structure.

### 3.8. FTIR Investigation

**Figure 13:**
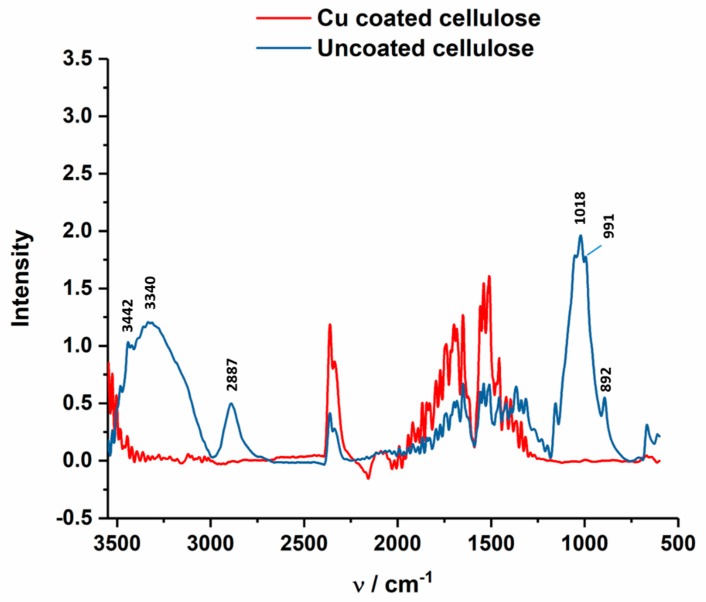
ATR-FTIR spectrum of Cu-coated cellulose (red) of the CU1 sample, which are compared to uncoated cellulose fabric (blue).

In Figure 13, ATR-FTIR spectrum of uncoated and Cu-coated cellulose is shown, which confirms the chemical changes of the Cu coating at the textile surface. The Cu-coated sample does not indicated characteristic cellulose peaks at 3442 cm^−1^ (O(2)H O(6) intramolecular hydrogen bond), 3340 cm^−1^ (O(3)H O(5) intramolecular hydrogen bond), 2887 cm^−1^ (C–H stretch), 1018 cm^−1^ (C–O vibration at C(3) O(3)H), 991 cm^−1^ (C–O valence vibration at C(6)) and 892 cm^−1^ (C–O–C valence vibration) [48].

It is believed that the coating do not affect the crystallinity of the cellulose structure because of the 0.5M NaOH concentration used during the electroless coating. In the work of Široký et al. molecular reorganization in the amorphous and quasi-crystalline areas is reported at 3.3 and 4.5 mol dm^−3^ of NaOH [48].

## 4. Conclusions

This work showed that it was possible to slow down the chemical reaction of the electroless copper deposition at the silver seeded CLY surface by using different concentration of Cu^2+^, CH_2_O, C_4_H_5_KO_6_, and NaOH. High Cu^2+^, C_4_H_5_KO_6_, NaOH, Tartrate, and CH_2_O concentrations led to a diffusion-controlled heterogeneous copper deposition and high homogeneous copper reaction in solution. The heterogeneous rate constant *k*_het_ was large comparing to the homogeneous rate constant *k*_hom_. Thus, the copper deposition on the cellulose surface did not increase further. The copper-coated CLY fabric was sensitive to mechanical deformations and humidity changes. Thus, it was able to transfer an electrical signal through the large surface area even at stretched and deformed condition by maintaining highly conductive due to the low resistance. The copper-coated CLY fabrics maintained conductivity after flex tests, cyclic tensile tests, during different RH, and duringwetting for 18 h (cycle 1) (Figure 12) in water (DI). After 6000 bending cycles, the copper layer was partially damaged but the fabric kept its conductivity by indicating a resistance of 6.6 Ω/1500 mm^2^. During cyclic elongation, the resistance of the copper-coated CLY textile decreased. At elongated state, the distance between copper-coated yarns decreased with an increase in contact area. The resistance of the copper-coated textile reached a similar value of 13 Ω/750 mm^2^ due to small elongation of 2% after cyclic elongation tests. After cyclic elongation and flex tests, the copper layer was still attached to the CLY substrate, which reveals its strong adhesion to the textile. Continuous swelling and loosening of copper-coated CLY textile during wetting and drying increased the fabrics resistance. The increase in resistance to 16 MΩ/375 mm^2^ can be related to damages in the Cu coating due to swelling and expansion of the CLY fabric. Fibrillation of the copper-coated CLY fabric was not observed according to LSM investigations.

## Figures and Tables

**Table 1:** Concentrations of chemicals used for electroless copper deposition.

Sample Name	C_4_H_5_KO_6_ (M)	NaOH (50 wt %) (M)	CuSO_4_ × 5 H_2_O (M)	CH_2_O (M)
CU 1	0.079	0.5	0.0284	0.94
CU 2	0.158	1	0.0568	1.88
CU 3	0.236	1.5	0.0852	2.82

**Table 2:** Summary of metallic content, moisture and conductivity in copper-coated cellulose lyocell (CLY) fabrics. Values defined as mean ± standard deviation.

Sample Name	[Ag] mg/g	*R* Ag Seed Fabric (Ω)	Cu Content mg/g	S_h_ µm	R_sh_ Ωsq^−1^	G_sh_ Ω^−1^	η Efficiency of the Process
CU 1	6.5 ± 0.5	1.3 × 10^11^	147 ± 0.3	2.75 ± 0.7	1.89 ± 0.9	0.66 ± 1.1	0.81
CU 2	14 ± 0.4	1.3 × 10^11^	234 ± 1.7	1.94 ± 0.3	5.24 ± 1.9	0.21 ± 0.1	0.65
CU 3	42 ± 1.0	1.3 × 10^11^	198 ± 0.2	1.54 ± 0.3	2.46 ± 2.0	0.62 ± 0.4	0.37

**Table 3:** Summary of the elemental distribution from the copper-coated cellulose (CU 1) fabric.

Element	Weight (%)	Atom (%)	Net. Int.	Error (%)
C (K)	7.40	24.60	66.92	11.67
O (K)	9.46	23.62	303.38	8.55
Ag (L)	1.77	0.66	51.29	13.88
Cu (K)	81.36	51.12	632.75	3.85
